# Early warning of bloodstream infection in elderly patients with circulating microparticles

**DOI:** 10.1186/s13613-021-00901-w

**Published:** 2021-07-13

**Authors:** Tingting Liu, Jiang Wang, Yaping Yuan, Jionghe Wu, Chao Wang, Yueqin Gu, Hongxia Li

**Affiliations:** 1 Department of Pulmonary and Critical Care Medicine, The Second Medical Center, National Clinical Research Center for Geriatric Diseases, Chinese PLA General Hospital, Beijing, 100853 China; 2 Centre of Pulmonary and Critical Care Medicine, Chinese PLA General Hospital, Beijing, 100853 China; 3 Department of Pulmonary and Critical Care Medicine, The Fourth Medical Center, Chinese PLA General Hospital, Beijing, 100853 China

**Keywords:** Microparticles, Elderly individuals, Bloodstream infection, Early warning model

## Abstract

**Background:**

The difficulty of early diagnosis of bloodstream infection in the elderly patients leads to high mortality. Therefore, it is essential to determine some new methods of early warning of bloodstream infection in the elderly patients for timely adjustment of treatment and improvement of prognosis.

**Methods:**

Patients aged over 65 years with suspected bloodstream infections were included and divided into bloodstream infection (BSI) and non-bloodstream infection (non-BSI) groups based on blood culture results. The morphology of microparticles (MPs) was observed by using transmission electron microscopy, and the number of MPs was dynamically monitored by flow cytometry.

**Results:**

A total of 140 patients were included in the study: 54 in the BSI group and 86 in the non-BSI group. Total MPs (T-MPs) ≥ 6000 events/µL (OR, 7.693; 95% CI 2.944–20.103, *P* < 0.0001), neutrophil-derived MPs (NMPs) ≥ 500 events/µL (OR, 12.049; 95% CI 3.574–40.623, *P* < 0.0001), and monocyte counts ≤ 0.4 × 10^9^/L (OR, 3.637; 95% CI 1.415–9.348, *P* = 0.007) within 6 h of fever were independently associated with bloodstream infection in the elderly patients. We also developed an early warning model for bloodstream infection in the elderly patients with an area under the curve of 0.884 (95% CI 0.826–0.942, *P* < 0.0001), sensitivity of 86.8%, specificity of 76.5%, positive predictive value of 70.8%, and negative predictive value of 89.8%.

**Conclusion:**

The early warning model of bloodstream infection based on circulating T-MPs, NMPs, and monocyte counts within 6 h of fever in the elderly patients was helpful in early detection of bloodstream infection and therefore promptly adjustment of treatment plan.

**Supplementary Information:**

The online version contains supplementary material available at 10.1186/s13613-021-00901-w.

## Introduction

As a critical illness, the morbidity and mortality of bloodstream infection increase with age. Studies have reported a mortality rate of 16%–49% for bloodstream infections in individuals aged 65–75 years and 21%–56% for those aged ≥ 75 years [1–3].The diagnosis of bloodstream infections relies on blood cultures, however, the long waiting time for blood cultures may delay the diagnosis. Non-culture-based diagnostic techniques, such as high-throughput sequencing, mass spectrometry, and real-time polymerase chain reaction techniques, are cumbersome, time-consuming, expensive, and difficult to promote [4–6]. C-reactive protein (CRP) and procalcitonin (PCT) are the most commonly used infection-related indicators in clinical practice, however too many influencing factors and poor sensitivity in the elderly population, limit their early diagnostic value [7, 8]. Thus, looking for certain suitable biomarkers for early warning of bloodstream infections in the elderly patients may reduce the mortality and morbidity of this critical condition.

Micropaticles (MPs) are submicron vesicles between 0.1 and 1 µm in diameter formed by cell membrane outgrowth and shedding upon activation or apoptosis, expressing parent cell-derived antigens [9]. MPs have a high expression of phosphatidylserine (PS) on their surface and carry proteins, lipids and nucleic acids [DNA, messenger RNA (mRNA), microRNA (miRNA)] from the mother cells [9]. MPs are derived from various types of circulating cells and involved in promoting coagulation, cell adhesion, chemotaxis, and release of various inflammatory factors and have been reported to contribute to the early diagnosis of sepsis-induced disseminated intravascular coagulation [10, 11]. However, to our knowledge, there are insufficient studies related to MPs and bloodstream infections in the elderly population, so this study was designed to explore the early warning role of circulating MPs on bloodstream infections in the elderly population.

## Materials and methods

### Patients

Patients aged over 65 years with suspected bloodstream infections from October 2018 to October 2020 at the Second Medical Center, Chinese PLA General Hospital, were included as the study group. The control group included elderly patients admitted for routine physical examinations during the same period. They presented with chronic diseases but were free of infectious disease. The inclusion criteria were: body temperature ≥ 38 °C with or without chills and (i) shock or decreased blood pressure (systolic blood pressure decreased by 40 mmHg or more than usual) or (ii) presence of central venous catheter or (iii) elevated infection index (leukocyte counts ≥ 15,000/µL or ≤ 4000/µL). The exclusion criteria were: end-stage disease with expected survival < 7 days; rheumatic autoimmune disease; malignancy (including hematologic tumors); abnormal liver function (Child–Pugh grade ≥ C); renal dysfunction (chronic kidney disease ≥ stage 3b); cardiac insufficiency (New York Heart Association ≥ grade 3); use of glucocorticoids or blood transfusion therapy 3 days before enrollment or during treatment; and failure to complete blood collection within 6 h and on the 2nd day of fever. According to the blood culture results, the patients were divided into non-bloodstream infection (non-BSI) group and bloodstream infection (BSI) group.

### Data collection

The basic information of the study subjects was collected, including sex, age, underlying disease (coronary heart disease, hypertension, diabetes, chronic obstructive pulmonary disease, hyperlipidemia, atrial fibrillation, chronic renal insufficiency), presence of indwelling lines (deep vein, urinary catheter, gastric tube), use of antibiotics within 30 days, infection status (site of infection, causative organism), and prognosis.

### Blood sampling

In the study group, leukocyte counts, neutrophil percentage, monocyte counts, CRP, and PCT levels were measured within 6 h, on days 2 and 14 of fever (except in patients who died), and 3 mL of venous blood was collected from the elbow of subjects in the study and control groups (on the day of enrollment) using a sodium citrate anticoagulation tube (3.2%, 0.109 M). To avoid the effect of punctured vessels on the MPs, blood samples for testing MPs were obtained at last.

MP isolation by differential centrifugation was performed: 3 mL of whole blood was centrifuged (4 °C, 2000*g*) for 10 min, and 1000 µL of supernatant was obtained to collect platelet-poor plasma (PPP); then, PPP was ultracentrifuged (4 °C, 13,000*g*) for 3 min, and 400 µL of supernatant was obtained to collect platelet-free plasma (PFP), which was stored in the refrigerator at − 80 °C.

### Microparticle analysis

#### Observation of MPs morphology by transmission electron microscopy

MPs (20 µl) from patients were placed on copper mesh and incubated for 5 min. We removed the remaining liquid with filter paper, and added 20 µl phosphotungstic acid. After standing for 10 min, any remaining liquid was removed with filter paper, and the sample was observed by transmission electron microscopy.

#### Detection of MPs number by flow cytometry

PFP was dissolved at room temperature and then ultracentrifuged (4 °C, 21,000*g*) for 60 min, and 100 µL of the bottom fraction was obtained as MPs. The number of MPs was detected by FACSCanto™ II flow cytometer (BD Bioscience, San Jose, CA, USA). Annexin V was used to identify the PS expressed on the surface of the MPs. Annexin V + MPs is the total number of MPs, CD11b + /Annexin V + represents leukocyte-derived MPs (LMPs), CD66b + /Annexin V + represents neutrophil-derived MPs (NMPs), CD14 + /Annexin V + represents monocyte-derived MPs (MMPs), and CD105 + /Annexin V + represents endotheliocyte-derived MPs (EMPs). The immunofluorescent monoclonal antibodies used were as follows (BioLegend, San Diego, CA, USA): Annexin V labeled by PE, CD11b labeled by APC-Cy7, CD66b labeled by FITC, CD14 labeled by APC-Cy7, and CD105 labeled by Percp-cy5.5. Moreover, 25 µL of MPs,1 µL Annexin V and 100 µL Annexin V binding buffer were added into two Eppendorf tubes A and B, and incubated for 20 min at room temperature, avoiding light. Then, 1 µL CD11b-APC-Cy7 and 1 µL CD66b-FITC were added into tube A, and 1 µL CD14-APC-Cy7 and 1 µL CD105-Percp-cy5.5 were added into tube B and incubated for 20 min at room temperature, avoiding light. Then, both tubes A and B were washed with phosphate-buffered saline (PBS), transferred to BD absolute counting tubes (BD Bioscience, San Jose, CA, USA), and tested on the machine. Furthermore, 0.2 µm, 0.5 µm, 1 µm, and 2 µm standard microspheres (Invitrogen, Carlsbad, CA, USA) were used to set the gate and determine the location of 0.1–1.0 µm MPs (Additional file 1: Fig. S1). To avoid the noise caused by dust and crystallization, the buffer and PBS were filtered through a 0.22-µm filter. MPs were counted in events/µL.

### Statistical analysis

IBM SPSS Statistics 23.0 (SPSS, Chicago, IL, USA) was used for statistical analyses. A Kolmogorov–Smirnov test was used to test the normality of the continuous variables. Quantitative data with normal distributions were expressed as the means and standard deviations and were analyzed by *t*-tests. Quantitative data with non-normal distributions were presented as the medians and interquartile ranges (IQRs) and were assessed with the Mann–Whitney U test. The Chi-square test or Fisher’s exact probability test was used to compare count data. Univariate analysis and multivariate logistic regression analysis (Forward: LR) were used to determine the independent influencing factors and an early warning model of bloodstream infection in elderly patients was established. The sensitivity and specificity of the early warning model were tested by receiver operating characteristic (ROC) curve. A *P* value less than 0.05 was considered statistically significant.

## Result

### MPs morphology

MPs had spherical structures with diameters ranging from 100 to 1000 nm, heterogeneity in size, and typical lipid bilayer structure (Additional file 1: Fig. S2).

### Basic clinical characteristics of the included patients

A total of 140 patients were included in this study: 86 patients in the non-BSI group and 54 patients in the BSI group (Additional file 1: Fig. S3). The rates of deep venous placement, urinary catheter retention, and antibiotic use within 30 days were significantly higher in the BSI group than those in the non-BSI group (92.6% vs 77.9%, *P* = 0.022; 48.1% vs 30.2%, *P* = 0.037; 79.6% vs 64%, *P* = 0.049, Additional file 2: Table S1).

In the BSI group, the infecting pathogens were mainly *Escherichia coli* in 17 (31.5%) cases, *Klebsiella pneumoniae* in 14 (25.9%) cases, *Pseudomonas aeruginosa* in 8 (14.8%) cases, and *Streptococcus haemolyticus* in 6 (11.1%) cases. In the non-BSI group, 60 (69.8%) cases were pulmonary infections, 15 (17.4%) cases were urinary tract infections, and five (5.8%) cases were biliary tract infections (Additional file 2: Table S1). The infecting pathogens were mainly *Klebsiella pneumoniae* in 21 (24.4%) cases, *Pseudomonas aeruginosa* in 20 (23.3%) cases, and *Escherichia coli* in 12 (14.0%) cases (Additional file 2: Table S2).

### Comparison and trends of inflammatory indexes in the two groups

The monocyte counts and percentage within 6 h of fever in the BSI group were significantly lower than those in the non-BSI group, and the PCT levels within 6 h and at the second day of fever in the BSI group were significantly higher than those in the non-BSI group (*P* < 0.05, Table 1).

**Table 1:** Inflammatory markers in non-BSI group and BSI group

Inflammatory markers	Group		P
	Non-BSI (n = 86)	BSI (n = 54)	
Within 6 h			
Leukocyte_1_, 10^9^/L, x ± s	9.79 ± 2.88	10.80 ± 3.50	0.085^*§*^
Neutrophil_1_, 10^9^/L, x ± s	8.25 ± 2.74	8.83 ± 3.41	0.300^*§*^
NP_1_,%, median (IQR)	0.83 [0.75, 0.89]	0.86 [0.78, 0.91]	0.086^*#*^
Monocyte_1_, 10^9^/L, median(IQR)	0.52 [0.35, 0.71]	0.38 [0.24, 0.59]	0.008^*#*^
MP_1_, %, median (IQR)	0.050 [0.035, 0.070]	0.040 [0.020, 0.063]	0.030^*#*^
CRP_1_, mg/L, median(IQR)	1.52 [0.78, 2.76]	1.92 [0.67, 4.74]	0.449^*#*^
PCT_1_, μg/L, median(IQR)	0.11 [0.05, 0.25]	0.22 [0.06, 0.45]	0.010^*#*^
Day 2			
Leukocyte_2_, 10^9^/L, x ± s	11.88 ± 4.90	11.74 ± 6.38	0.886^*§*^
Neutrophil_2_, 10^9^/L, x ± s	9.66 ± 4.28	9.68 ± 5.87	0.999^*§*^
NP_2_, %, median(IQR)	0.82 [0.75, 0.87]	0.83 [0.75, 0.87]	0.922^*#*^
Monocyte_2_, 10^9^/L, median(IQR)	0.56 [0.42, 0.80]	0.47 [0.30, 0.82]	0.210^*#*^
MP_2_, %, median(IQR)	0.051 [0.039, 0.077]	0.050 [0.033, 0.076]	0.513^*#*^
CRP_2_, mg/L, median(IQR)	9.99 [6.96, 15.35]	12.44 [6.04, 16.80]	0.636^*#*^
PCT_2_, μg/L, median(IQR)	0.75 [0.20, 2.15]	3.45 [1.28, 13.14]	< 0.0001^*#*^
Day 14			
Leukocyte_3_, 10^9^/L, x ± s	6.51 ± 1.70	6.53 ± 1.55	0.739^*§*^
Neutrophil_3_, 10^9^/L, x ± s	4.34 ± 1.46	4.29 ± 1.29	0.674^*§*^
NP_3_, %, median (IQR)	0.65 [0.59, 0.70]	0.66 [0.58, 0.74]	0.554^*#*^
Monocyte_3_, 10^9^/L, median (IQR)	0.49 [0.33, 0.64]	0.47 [0.35, 0.58]	0.977^*#*^
MP_3_, %, median (IQR)	0.074 [0.059, 0.092]	0.069 [0.056, 0.091]	0.541^*#*^
CRP_3_, mg/L, median (IQR)	0.92 [0.47, 1.52]	0.93 [0.62, 1.59]	0.418^*#*^
PCT_3_, μg/L, median (IQR)	0.07 [0.05, 0.12]	0.12 [0.05, 0.20]	0.129^*#*^

*BSI* bloodstream infection, *Non-BSI* non-bloodstream infection, *NP* neutrophil percentage, *MP* monocyte percentage, *CRP* C-reactive protein, *PCT* procalcitonin

_1_: within 6 h; _2_: day 2; _3_: day 14

^§^t test

^#^Mann–Whitney U test

Neutrophil percentage peaked within 6 h of fever in both groups and was significantly higher in the BSI group within 6 h of fever than that on the next day (*P* = 0.049). In the BSI group, monocyte counts peaked on day 2 and were significantly lower within 6 h of fever than those on day 2 (*P* = 0.029) and showed no difference between day 2 and 14 (*P* = 0.471). In the non-BSI group, the monocyte counts showed no difference within 6 h of fever and on day 2 (*P* = 0.188) and were significantly higher on day 2 than those on day 14 (*P* = 0.010). CRP and PCT levels peaked on day 2 of fever in both groups and were significantly higher than those within 6 h of fever (*P* < 0.05). All inflammatory indexes (except monocyte counts in BSI group) significantly decreased on day 14.

### Comparison and trends of MPs in the two groups

**Fig. 1:**
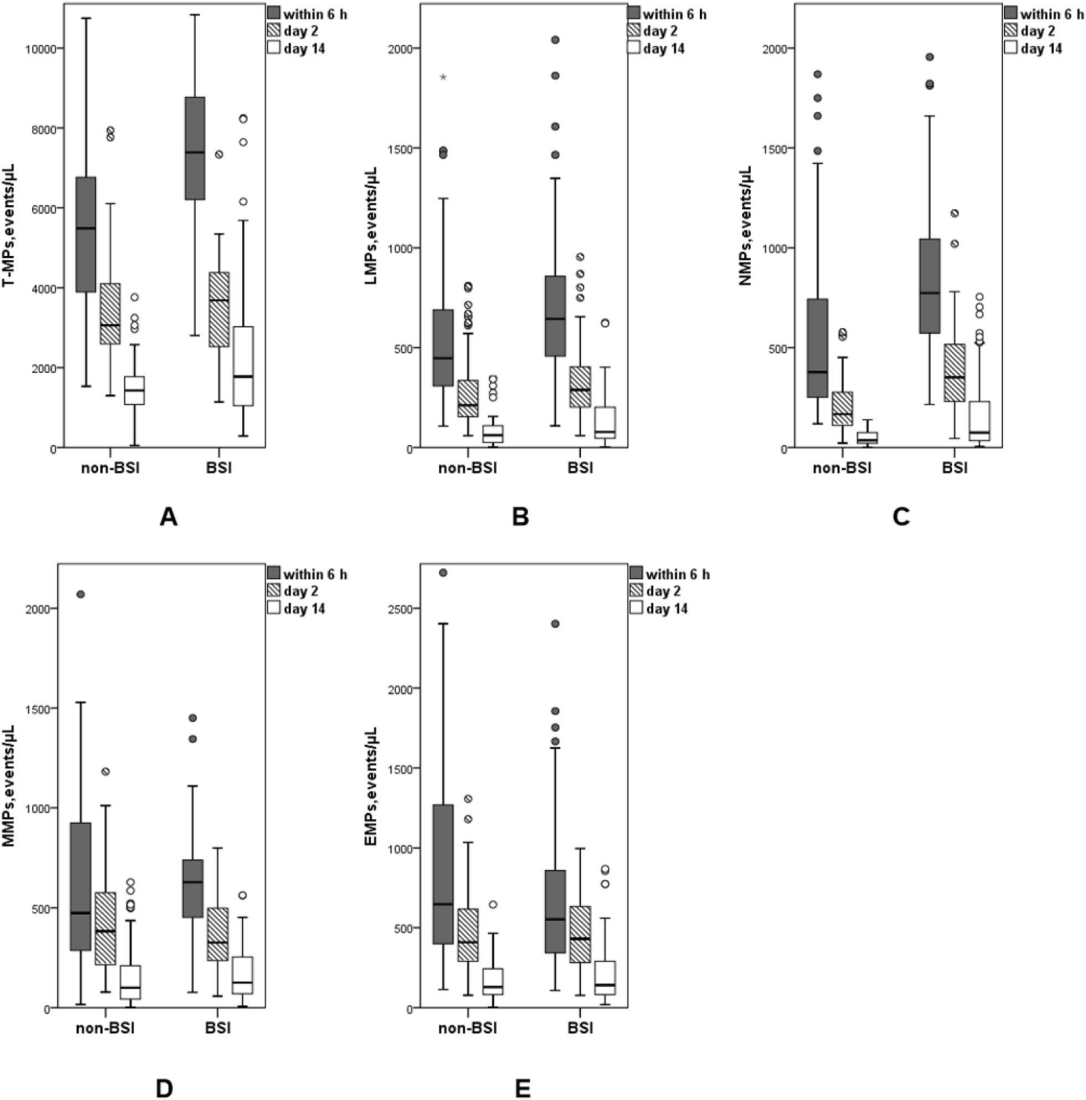
Dynamic monitoring of MPs. T-MPs, LMPs, NMPs, MMPs, and EMPs peaked within 6 h of fever in both groups and were significantly higher than those on day 2. All types of MPs decreased gradually with time. **A** Total MPs, **B** leukocyte-derived MPs, **C** neutrophil-derived MPs, **D** monocyte-derived MPs, and **E** endotheliocyte-derived MPs. *BSI: bloodstream infection; non-BSI: non-bloodstream infection; MPs: microparticles; T-MPs: total microparticles; LMPs: leukocyte-derived microparticles; NMPs: neutrophil-derived microparticles; MMPs: monocyte-derived microparticles; EMPs: endotheliocyte-derived microparticles*

Within 6 h of fever and on day 2, total MP (T-MP), LMP, and NMP levels were significantly higher in the BSI group than those in the non-BSI group, and there was no difference in MMP and EMP levels between the two groups (Table 2). On day 14, NMP levels were significantly higher in the BSI group than those in the non-BSI group (*P* = 0.016, Table 2, Fig. 1, Additional file 1: Fig. S4).

**Table 2:** Microparticles in non-BSI group and BSI group

MPs, events/μL, median (IQR)	Group		P
	Non-BSI (n = 86)	BSI (n = 54)	
Within 6 h			
T-MPs_1_	5487 [3880, 6815]	7386 [6182, 8861]	< 0.0001^*#*^
LMPs_1_	447 [308, 696]	644 [450, 867]	0.004^*#*^
NMPs_1_	378 [247, 744]	773 [568, 1048]	< 0.0001^*#*^
MMPs_1_	474 [286, 953]	628 [451, 760]	0.118^*#*^
EMPs_1_	648 [386, 1271]	553 [323, 863]	0.306^*#*^
Day 2			
T-MPs_2_	3058 [2580, 4108]	3681 [2509, 4397]	< 0.0001^*#*^
LMPs_2_	212 [152, 337]	287 [202, 415]	0.019^*#*^
NMPs_2_	167 [110, 280]	352 [230, 519]	< 0.0001^*#*^
MMPs_2_	382 [214, 576]	326 [205, 499]	0.334^*#*^
EMPs_2_	410 [288, 621]	432 [280, 637]	0.868^*#*^
Day 14			
T-MPs_3_	1151 [612, 1603]	1124 [735, 2656]	0.301^*#*^
LMPs_3_	66 [26, 115]	78 [46, 230]	0.082^*#*^
NMPs_3_	43 [22, 81]	75 [34, 257]	0.016^*#*^
MMPs_3_	48 [31, 89]	63 [34, 250]	0.096^*#*^
EMPs_3_	56 [38, 94]	62 [47, 116]	0.167^*#*^

*BSI* bloodstream infection, *Non-BSI* non-bloodstream infection, *MPs* microparticles, *T-MPs* total microparticles, *LMPs* leukocyte-derived microparticles, *NMPs* neutrophil-derived microparticles, *MMPs* monocyte-derived microparticles, *EMPs* endotheliocyte-derived microparticles

_1_: within 6 h; _2_: day 2; _3_: day 14

^#^Mann–Whitney U test

T-MP, LMP, NMP, MMP, and EMP levels peaked within 6 h of fever in both groups and gradually decreased with time (Table 2, Fig. 1).

### Comparison of MPs in the study group on day 14 with the control group

After excluding patients who died within 14 days (*n* = 8), T-MPs were significantly higher in the study group on day 14 than those in the control group, and LMPs and NMPs were significantly higher in the BSI group than those in the control group, while there was no significant difference in all types of MPs between the non-BSI group and control group (Additional file 2: Table S3). There was no significant difference in clinical characteristics between study group and control group (Additional file 2: Table S3).

### Establishment of bloodstream infection early warning model in the elderly population

Factors that differed within 6 h of fever (*P* < 0.1) in the univariate analysis were included in the multivariate logistic regression analysis (forward: LR), and T-MPs ≥ 6000 events/µL (OR, 7.693; 95% CI 2.944–20.103, *P* < 0.0001), NMPs ≥ 500 events/µL (OR, 12.049; 95% CI 3.574–40.623, *P* < 0.0001), and monocyte counts ≤ 0.4 × 10^9^/L (OR, 3.637; 95% CI 1.415–9.348, *P* = 0.007) were independently associated with bloodstream infection in the elderly population (Table 3).

**Table 3:** Multivariate logistic regression analysis for early warning of bloodstream infection in the elderly

Variables	β	S.E	Wald	df	Sig	OR	95% CI for OR	
							Lower	Upper
T-MPs_1_, ≥ 6000 events/μL	2.040	0.490	17.332	1	< 0.0001	7.693	2.944	20.103
NMPs_1_, ≥ 500 events/μL	2.489	0.620	16.110	1	< 0.0001	12.049	3.574	40.623
Monocyte counts_1_, ≤ 0.4 × 10^9^/L	1.291	0.482	7.185	1	0.007	3.637	1.415	9.348
Constant	− 3.977	0.713	31.137	1	< 0.0001	0.019		

*T-MPs*: total microparticles, *NMPs* neutrophil-derived microparticles

_1_: within 6 h

Based on the results of multivariate regression analysis, an early warning model of bloodstream infection in the elderly population was established: Logit(P) = − 3.977 + 2.040 × T-MPs_1_(≥ 6000 events/µL = 1, < 6000 events/µL = 0) + 2.489 × NMPs_1_(≥ 500 events/µL = 1, < 500 events/µL = 0) + 1.291 × monocyte counts_1_ (≤ 0.4 × 10^9^/L = 1, > 0.4 × 10^9^/L = 0).

It was further transformed to obtain: Y = 1.580 × T-MPs_1_(≥ 6000 events/µL = 1, < 6000 events/µL = 0) + 1.928 × NMPs_1_(≥ 500 events/µL = 1, < 500 events/µL = 0) + monocyte counts_1_(≤ 0.4 × 10^9^/L = 1, > 0.4 × 10^9^/L = 0).

### Receiver operating characteristic (ROC) curve to evaluate the predictive value of early warning model for predicting bloodstream infection in the elderly population

**Fig. 2:**
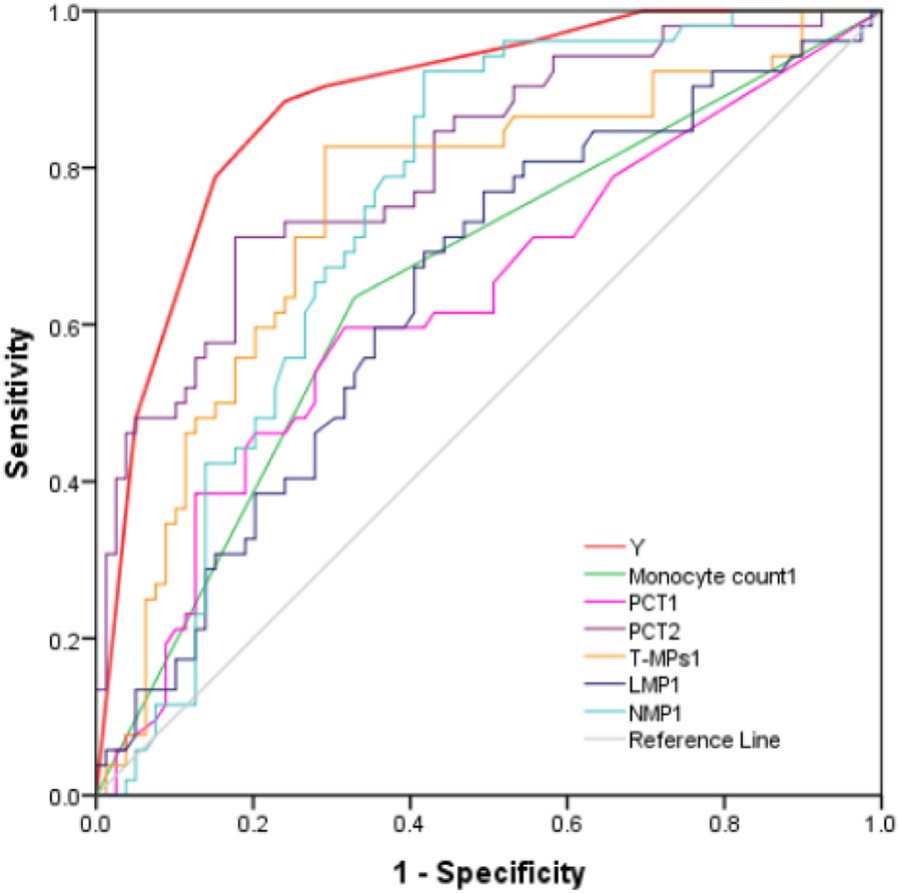
Receiver operating characteristic (ROC) curve to evaluate the predictive value of early warning model and other factors for predicting bloodstream infection in the elderly population. Area under the curve of warning model was 0.884 (95%CI 0.826–0.942, P < 0.0001). *PCT: procalcitonin; T-MPs: total microparticles; LMPs: leukocyte-derived microparticles; NMPs: neutrophil-derived microparticles; *_*1*_*: within 6 h; *_*2*_*: day 2*

The ROC curve showed that the area under the curve (AUC) of the early warning model for predicting bloodstream infection in the elderly population was 0.884 (95% CI 0.826–0.942, *P* < 0.0001), the optimal cutoff value of *Y* was 3.5, the sensitivity was 86.8%, the specificity was 76.5%, the positive predictive value was 70.8%, and the negative predictive value was 89.8% (Table 4, Fig. 2).

**Table 4:** Predictive performance of early warning model and other factors for predicting bloodstream infection in the elderly population

Variables	Cutoff	AUC 95% CI	Sensitivity	Specificity	PPV	NPV	P
Y	3.5	0.884(0.826, 0.942)	86.8%	76.5%	70.8%	89.8%	< 0.0001
Monocyte counts_1_, ≤ 0.4 × 10^9^/L		0.645(0.548, 0.741)	62.3%	66.7%	55.0%	73.0%	0.005
PCT_1_, μg/L	0.185	0.630(0.530, 0.729)	60.4%	67.9%	55.2%	72.4%	0.012
PCT_2_, μg/L	2.76	0.807(0.731, 0.884)	69.8%	82.1%	71.2%	81.2%	< 0.0001
T-MPs_1_, events/μL	6000	0.751(0.663, 0.839)	81.1%	69.9%	63.2%	85.3%	< 0.0001
LMPs_1_, events/μL	450	0.642(0.546, 0.739)	75.5%	51.8%	50.0%	76.8%	0.006
NMPs_1_, events/μL	500	0.748(0.664, 0.831)	92.5%	57.8%	58.3%	92.3%	< 0.0001

*PCT* procalcitonin, *MPs* microparticles, *T-MPs* total microparticles, *LMPs* leukocyte-derived microparticles, *NMPs* neutrophil-derived microparticles, *PPV* positive predictive value, *NPV* negative predictive value

_*1*_*: within 6 h; *_*2*_*: day 2*

## Discussion

MPs exert pro-inflammatory, procoagulant, and thrombogenic effects by stimulating receptors on the surface of target cells or transferring substances, such as proteins, lipids, mRNAs, or microRNAs, that they carry to target cells [12]. MPs have been shown to be associated with infection [11, 13, 14], and we also found that MPs of all cellular origins were significantly increased within 6 h of infection in elderly patients and gradually decreased with treatment and improvement of infection, suggesting that MPs are associated with the occurrence and prognosis of infection. More importantly, we found that T-MPs and NMPs combined with monocyte counts within 6 h of fever in elderly patients could provide early warning of bloodstream infection, which could help adjust treatment early and reduce mortality.

We included elderly patients who presented with high fever with or without chills during hospitalization, all of whom were suspected to have bloodstream infection from the clinical presentation, and 38.6% (54/140) of the patients were finally diagnosed in the subsequent observation. Although the disease severity at enrollment was similar to that of patients with non-bloodstream infections, approximately one-third of these patients died within 30 days of infection. A series of studies have indicated that bloodstream infections can cause septic shock, leading to multiorgan dysfunction and significantly higher mortality [15, 16], as does delayed antibiotic administration [17]. Therefore, early diagnosis and treatment are key to improvement of prognosis of bloodstream infections [18, 19]. It has been reported that PCT level rapidly increases in the early stages of infection (within 6 h) and can indicate bloodstream infection [20]. However, in the elderly population, we found that PCT can indicate bloodstream infection on day 2 of fever (Table 4), when the optimal time for treatment of bloodstream infection has been missed. A series of studies also suggest that PCT has extremely limited value in early warning of bloodstream infection in elderly patients [7, 8]. Therefore, rapid and effective diagnostic markers are still lacking.

In the present study, circulating T-MPs, and NMPs within 6 h of fever provided early warning of bloodstream infection, which was able to identify bloodstream infection significantly earlier than PCT [20], although there was no significant difference in circulating leukocyte and neutrophil counts between the two groups at this time. Nieuwland et al. [21] have also found that early circulating platelet-derived MP (PMP) and NMP levels in patients with septicemia caused by *Streptococcus meningitidis* were significantly increased compared with health donors. Timar et al. [13] found that, in patients with *Staphylococcus aureus* bacteremia, circulating NMP levels were significantly increased at 24 h of fever compared with health volunteers. However, these above-mentioned studies did not compare the early warning effect of MPs and other inflammatory markers and did not dynamically monitor the changes in MPs. When we dynamically monitored circulating MPs, we found that T-MP, LMP, and NMP levels were still higher in the BSI group than those in the non-BSI group on day 2 of fever, and levels of all types of MPs decreased significantly on day 14 of fever, but NMP levels were still significantly higher in the BSI group than those in the non-BSI group. This suggests that, during bloodstream infection, pathogenic microorganisms may rapidly induce the release of MPs from circulating cells through direct stimulation or their by-products, and this effect may persist until the late stages of infection. In fact, we compared the MPs in patients who were survived and died within 30 days. We found that the level of various MPs within 6 h of fever in the death group was significantly higher than those in the non-death group. However on day 2, the level of MPs in both groups decreased significantly, and there was no difference between the two groups. On day 14, the level of MPs in the death group was significantly higher than that in the non-death group again, suggesting that the level of MPs might predict the prognosis. In a study by Delabranche et al. [11], LMP levels were significantly elevated on the day of admission in patients with septic shock and significantly decreased as their condition improved. O'Dea et al. [14] found that, in patients with massive burns with infection, LMP and NMP levels were significantly elevated during the phase of systemic inflammatory syndrome and significantly decreased after administration of anti-infection and other treatments. These studies suggest that MPs are associated with the occurrence and regression of infection.

Moreover, we found that circulating monocyte counts and monocyte percentage within 6 h of fever in the BSI group were significantly lower than those in the non-BSI group, which is an interesting phenomenon. As precursor cells of macrophages, monocytes are capable of cytokine production, pathogen clearance, and antigen presentation [22, 23] and may be rapidly activated to eliminate pathogenic bacteria in primary bloodstream infections, but the bloodstream infections in this study were mostly secondary, and monocytes may have been recruited at the foci of primary infection, and neutrophil levels were more rapidly elevated in bloodstream infections, so the number of monocytes was relatively low. Actually after adjusting the number of MPs to their respective parent cells, We found MMPs/Monocyte were significantly higher in the BSI group than those in the non-BSI group within 6 h of fever, although Monocyte counts were lower in the BSI group at this time. This suggests that monocytes are activated to produce more MMPs within 6 h of fever during bloodstream infection.

By establishing an early warning model for bloodstream infections in the elderly population based on the results of multivariate analysis, we can diagnose 86.8% of bloodstream infections quickly and accurately, so as to guide clinicians to effectively fight infection and improve patient prognosis. In addition, based on this model, when a subject is tested negative, the correct rate of excluding bloodstream infection is 89.8%, which can avoid abuse of broad-spectrum antibiotics that induce bacterial resistance and promote the rational use of antibiotics.

There are some drawbacks in this study. First, the number of cases of Gram-positive bacterial and fungal bloodstream infections was small, therefore, no correlation could be drawn between etiology and MP production. Second, this is a single-center study with a small sample size. Third, platelet-derived MPs (PMPs) represent the majority of circulating total MPs, however, in the pre-experiment, we found that PMPs (represented by CD41 + /Annexin V +) were not closely related to infection, on the other hand some studies showed that PMPs were more closely related to thrombosis [24], angiogenesis [25], and tumor progression [26, 27], therefore, we did not monitor PMPs.

## Conclusion

Early diagnosis and treatment of bloodstream infections can improve patient prognosis and reduce the financial burden on patients and hospitals. We found for the first time that circulating T-MPs and NMPs within 6 h of fever can independently provide early warning of bloodstream infection in the elderly population, and combined with monocyte counts within 6 h of fever, an early warning model of bloodstream infection was established. Moreover, dynamic monitoring of circulating MPs can help determine the severity of infection and prognosis. This provides a new method for early identification of bloodstream infections and dynamic monitoring of infection regression. Whether MPs have the same predictive value for bloodstream infections in young adults deserves further exploration.

## Supplementary Information

**Additional file 1: **

**Additional file 2: **

## Data Availability

The data used and/or analyzed during the current study are available from the corresponding author on reasonable request.
