# Supplementary material for: Early warning of bloodstream infection in elderly patients with circulating microparticles
Source: Ann Intensive Care. 2021 Jul 13;11:110. doi: 10.1186/s13613-021-00901-w (PMC8276897; doi:10.1186/s13613-021-00901-w)
Supplement: Supplementary file 1 — Additional file 1: [file 13613_2021_901_MOESM1_ESM.docx]

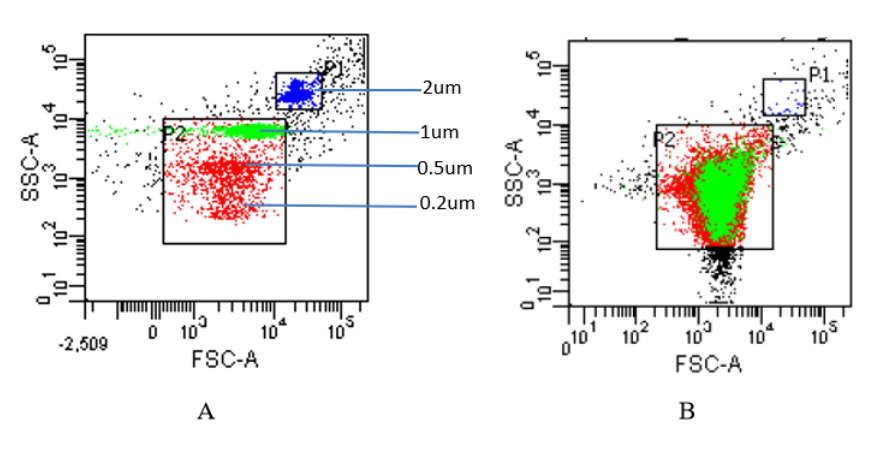


**Figure S1** Setting of gates in the flow cytometer. The gates in A represented the positions of particles with diameters of 2 µm, 1 µm, 0.5 µm, 0.2µm respectively, and P2 was the position of particles with diameters of 0.1-1 µm, that was, the position of the microparticles (MPs). B showed the flow diagram of MPs on the tester, and most MPs were in the P2 gate.


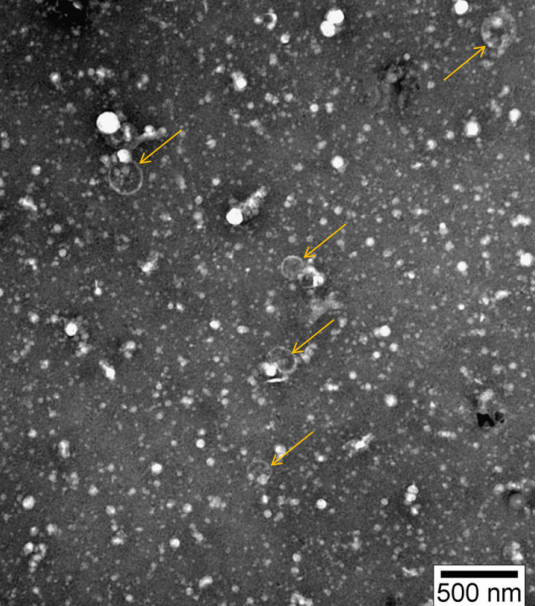


**Figure S2**  The morphology of microparticles (MPs) was observed using transmission electron microscopy. MPs had spherical structures under electron microscopy, with diameters ranging from 100 to 1000 nm, heterogeneity in size, and typical lipid bilayer structure.


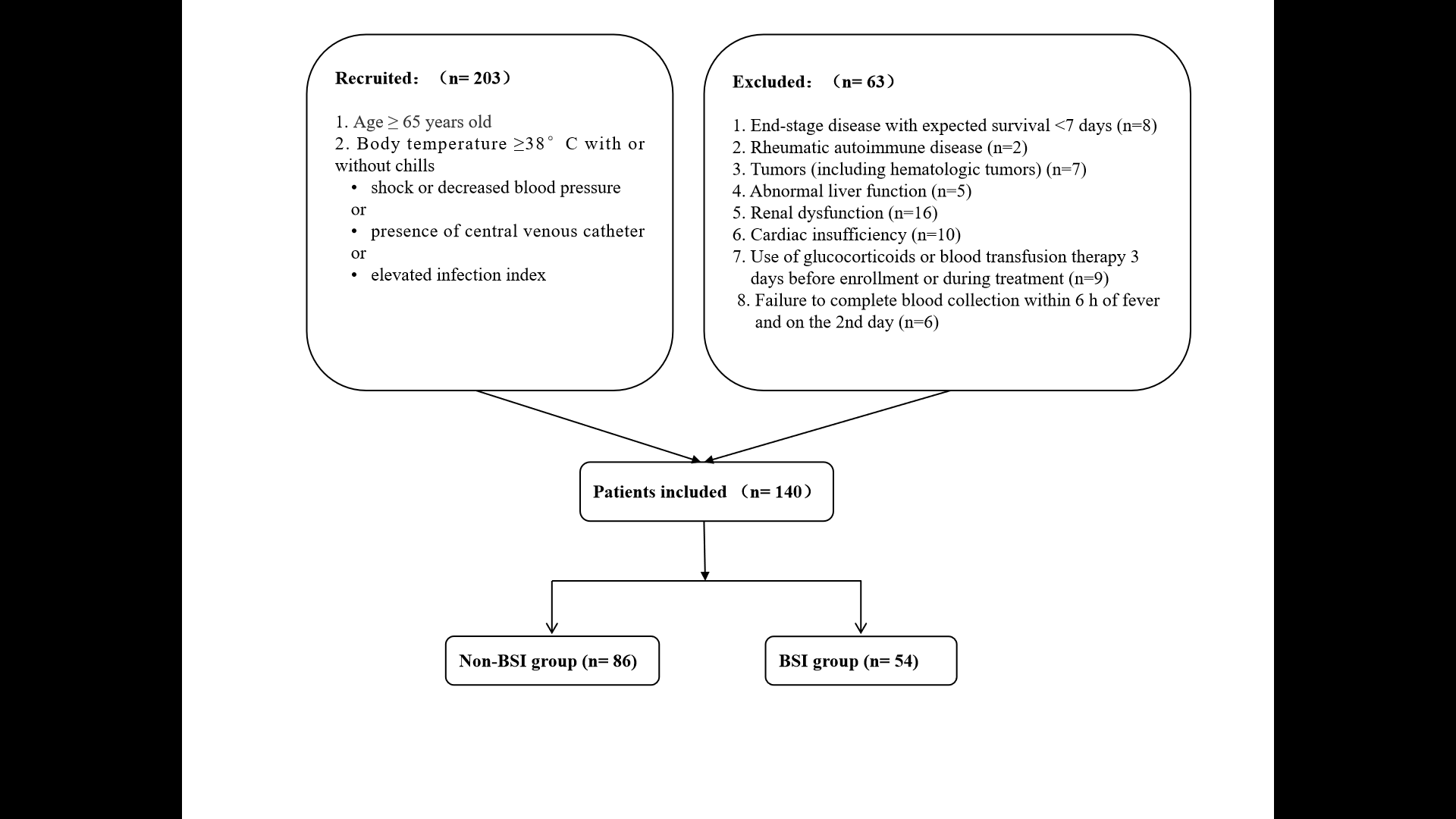


**Figure S3**  Screening of patients selection and enrollment.


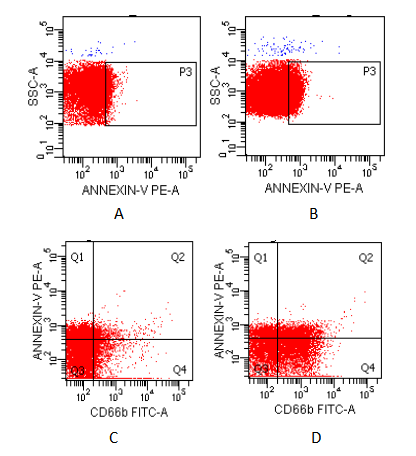


**Figure S4** MPs were displayed in Flow Cytometer. A showed total MPs in non-BSI group, B showed total MPs in BSI group; C showed NMPs in non-BSI group and D showed NMPs in BSI group. *BSI: Bloodstream infection; non-BSI: Non-bloodstream infection; MPs: Microparticles; NMPs: Neutrophil-derived microparticles.*
