# Supplementary material for: Early warning of bloodstream infection in elderly patients with circulating microparticles
Source: Ann Intensive Care. 2021 Jul 13;11:110. doi: 10.1186/s13613-021-00901-w (PMC8276897; doi:10.1186/s13613-021-00901-w)
Supplement: Supplementary file 2 — Additional file 2: [file 13613_2021_901_MOESM2_ESM.docx]

Table S1 Clinical characteristics and Laboratory findings of 140 enrolled patients

|  | | All patients  (n=140) | Groups | | P |
| --- | --- | --- | --- | --- | --- |
|  |  |  | non-BSI（n=86） | BSI（n=54） |  |
| Characteristics | |  |  |  |  |
| Age, year, x±s | | 90.56±6.06 | 90.88±6.20 | 90.04±5.84 | 0.677*^§^* |
| Gender, male, N (%) | | 129(92.1) | 80(93.0) | 49(90.7) | 0.625*^*^* |
| Underlying disease, N (%) | |  |  |  |  |
|  | COPD | 57(40.7) | 34(39.5) | 23(42.6) | 0.720*^*^* |
|  | Hypertension | 95(67.9) | 59(68.6) | 36(66.7) | 0.811*^*^* |
|  | Coronary heart disease | 95(67.9) | 55(64.0) | 40(74.1) | 0.212*^*^* |
|  | Atrial fibrillation | 51(36.4) | 31(36.0) | 20(37.0) | 0.906*^*^* |
|  | Hyperlipidemia | 56(40.0) | 34(39.5) | 22(40.7) | 0.887*^*^* |
|  | Diabetes mellitus | 47(33.6) | 31(36.0) | 16(29.6) | 0.434*^*^* |
|  | Chronic kidney disease | 23(16.4) | 11(12.8) | 12(22.2) | 0.143*^*^* |
|  | Neurological disease | 46(32.9) | 28(32.6) | 18(33.3) | 0.924*^*^* |
| Invasive ventilation, N (%) | | 72(51.4) | 40(46.5) | 32(59.3) | 0.142*^*^* |
| Deep vein catheterization, N (%) | | 117(83.6) | 67(77.9) | 50(92.6) | 0.022*^*^* |
| Urinary catheter, N (%) | | 52(37.1) | 26(30.2) | 26(48.1) | 0.037*^*^* |
| Stomach tube, N (%) | | 114(81.4) | 69(80.2) | 45(83.3) | 0.646*^*^* |
| History of antibiotic use within 30 days, N (%) | | 98(70.0) | 55(64.0) | 43(79.6) | 0.049*^*^* |
| Infection site, N (%) | |  |  |  |  |
|  | bloodstream infection | 54(38.6) | 0 | 54(100) |  |
|  | pulmonary infection | 60(42.9) | 60(69.8) | 0 |  |
|  | urinary system infection | 15(10.7) | 15(17.4) | 0 |  |
|  | Biliary tract infection | 5(3.6) | 5(5.8) | 0 |  |
|  | Others | 6(4.3) | 6(7.0) | 0 |  |
| Use of vasoactive drugs, N (%) | | 19(13.6) | 12(14.0) | 7(13.0) | 0.868*^*^* |
| 30-day mortality, N (%) | | 32(22.9) | 16(18.6) | 16(29.6) | 0.130*^*^* |
| Laboratory findings | |  |  |  |  |
|  | Albumin , g/L,x±s |  | 32.87±4.24 | 33.02±4.24 | 0.604*^§^* |
|  | Creatinine, μmol/L,median(IQR) |  | 75.0[53.0,124.3] | 84.5[61.5,145.8] | 0.140*^#^* |
|  | Bilirubin, μmol/L, x±s |  | 15.41±13.47 | 13.39±9.11 | 0.397*^§^* |
|  | ALT, U/L, median (IQR) |  | 16.0[10.0,25.3] | 15.0[10.8,28.7] | 0.939*^#^* |
| SOFA, median(IQR) | |  | 5[3,7] | 6[4,9] | 0.241*^#^* |

*BSI: Bloodstream infection; non-BSI: Non-bloodstream infection; COPD, Chronic obstructive pulmonary disease; ALT, Alanine aminotransferase;* *SOFA, Sequential organ failure assessment*

*^*^Chi-square test*

*^§^t test*

*^#^Mann-Whitney U test*

Table S2 Pathogenic bacteria and antibiotics of 140 enrolled patients

|  | | All patients  (n=140) | Groups | |
| --- | --- | --- | --- | --- |
|  |  |  | non-BSI（n=86） | BSI（n=54） |
| Pathogenic bacteria, N (%) | |  |  |  |
|  | Escherichia coli | 29(20.7) | 12(14.0) | 17(31.5) |
|  | Klebsiella pneumoniae | 35(25.0) | 21(24.4) | 14(25.9) |
|  | Pseudomonas aeruginosa | 28(20.0) | 20(23.3) | 8(14.8) |
|  | Streptococcus hemolyticus | 6(4.3) | 0 | 6(11.1) |
|  | Burkholderia cepacia | 4(2.9) | 1(1.2) | 3(5.6) |
|  | Staphylococcus aureus | 2(1.4) | 0 | 2(3.7) |
|  | Enterococcus faecalis | 6 | 5(5.8) | 1(1.9) |
|  | [Serratia](D:/Dict/8.9.6.0/resultui/html/index.html#/javascript:;) [marcescens](D:/Dict/8.9.6.0/resultui/html/index.html#/javascript:;) | 2(1.4) | 1(1.2) | 1(1.9) |
|  | Acinetobacter baumannii | 7(5.0) | 7(8.1) | 0 |
|  | Stenotrophomonas maltophilia | 5(3.6) | 5(5.8) | 0 |
|  | Proteus mirabilis | 2(1.4) | 2(2.3) | 0 |
|  | Candida albicans | 1(0.7) | 0 | 1(1.9) |
|  | Candida parapsilosis | 1(0.7) | 0 | 1(1.9) |
|  | Others | 12(8.6) | 12(14.0) | 0 |
| Antibiotics, N (%) | |  |  |  |
|  | Carbapenem monotherapy | 51(36.4) | 34(39.5) | 17(31.5) |
|  | Carbapenems + Tigecycline | 37(26.4) | 25(29.1) | 12(22.2) |
|  | Carbapenems + Vancomycin | 8(5.7) | 2(2.3) | 6(11.1) |
|  | Carbapenems + Levofloxacin | 8(5.7) | 5(5.8) | 3(5.6) |
|  | Cephalosporin monotherapy | 17(12.1) | 11(12.8) | 6(11.1) |
|  | Cephalosporin + Tigecycline | 11(7.9) | 5(5.8) | 6(11.1) |
|  | Cephalosporin + Levofloxacin | 6(4.3) | 4(4.7) | 2(3.7) |
|  | Cephalosporin + Caspofungin | 2(1.4) | 0 | 2(3.7) |

*BSI: Bloodstream infection; non-BSI: Non-bloodstream infection*

Table S3 Comparison of microparticles and clinical characteristics in the study group on day 14 with control group

|  |  | non-BSI(n=81) | BSI(n=51) | Control Group  (n=20) | P*^&^* | P*^$^* |
| --- | --- | --- | --- | --- | --- | --- |
| Age, year, x±s | | 90.72±6.29 | 90.12±6.00 | 89.30±7.68 | 0.391*^§^* | 0.635*^§^* |
| Gender, male,N (%) | | 76(93.8) | 48(94.1) | 19(95.0) | 0.842*^*^* | 0.885*^*^* |
| Underlying disease, N (%) | |  |  |  |  |  |
|  | COPD | 32(39.5) | 23(45.1) | 6(30.0) | 0.432*^*^* | 0.244*^*^* |
|  | Hypertension | 55(67.9) | 34(66.7) | 11(55.0) | 0.278*^*^* | 0.359*^*^* |
|  | Coronary heart disease | 51(63.0) | 39(76.5) | 12(60.0) | 0.806*^*^* | 0.165*^*^* |
|  | Atrial fibrillation | 30(37.0) | 19(37.3) | 6(30.0) | 0.556*^*^* | 0.565*^*^* |
|  | Hyperlipidemia | 34(42.0) | 20(39.2) | 8(40.0) | 0.872*^*^* | 0.951*^*^* |
|  | Diabetes mellitus | 29(35.8) | 14(27.5) | 7(35.0) | 0.947*^*^* | 0.531*^*^* |
|  | Chronic kidney disease | 9(11.1) | 11(21.6) | 2(10.0) | 1.000*^*^* | 0.326*^*^* |
| MPs, events/μL | |  |  |  |  |  |
|  | T-MPs | 1427[1070,1772] | 1776[1026,3060] | 587[485,716] | ＜0.0001*^#^* | ＜0.0001*^#^* |
|  | LMPs | 66[26,115] | 78[46,230] | 56[33,66] | 0.321*^#^* | 0.023*^#^* |
|  | NMPs | 43[22,81] | 75[34,257] | 32[22,59] | 0.221*^#^* | 0.004*^#^* |
|  | MMPs | 48[31,89] | 63[34,250] | 50[32,68] | 0.539*^#^* | 0.179*^#^* |
|  | EMPs | 56[48,94] | 62[47,116] | 58[46,76] | 0.418*^#^* | 0.239*^#^* |

*BSI: Bloodstream infection; non-BSI: Non-bloodstream infection; MPs: Microparticles; T-MPs: Total microparticles; LMPs: Leukocyte-derived microparticles; NMPs: Neutrophil-derived microparticles; MMPs: Monocyte-derived microparticles; EMPs: Endotheliocyte-derived microparticles*

*^&^non-BSI vs Control Group*

*^$^BSI vs Control Group*

*^*^Chi-square test*

*^§^t test*

*^#^Mann-Whitney U test*
